# Supplementary material for: Attitude and knowledge of healthcare providers towards female genital cosmetic surgery: A cross-sectional study
Source: PLoS One. 2026 Mar 20;21(3):e0343634. doi: 10.1371/journal.pone.0343634 (PMC13004357; doi:10.1371/journal.pone.0343634)
Supplement: Attachment — Submitted filename: Response_FGCS.docx [file pone.0343634.s002.docx]

Manuscript Title: ***Attitude and Knowledge of Healthcare Providers Toward Female Genital Cosmetic Surgery: A Cross-Sectional Study***

We would like to thank the Academic Editor and the reviewers for their thoughtful comments and suggestions, which have helped us substantially improve our manuscript. Below, we provide point-by-point responses to each comment. Revisions have been made accordingly in the revised manuscript.

**Editorial and Journal Requirements**

**1. English Language and Style**

*Editor: A general revision of the English language is necessary to make the article more straightforward. I found many spelling mistakes that need to be corrected.*

**Response:**

Thank you for your observation. We have thoroughly revised the manuscript for spelling, grammar, and clarity. The revised version improves flow and ensures consistency in scientific language, capitalization, tense, and punctuation. A clean and tracked version has been uploaded.

**2. Disparity in Specialties**

*There is a significant disparity in the specialties of the participants. This should be addressed as it could lead to a different evaluation of the results.*

**Response:**

We agree. We have now explicitly acknowledged this as a limitation in the Discussion and Strengths and Limitations sections. We also clarified the composition of participant groups in the Results section.

**3. Limited Number of FGCS Providers**

*Only 27 participants admit to performing FGCS "several times". This must be emphasized... consider focusing on these participants for subgroup analysis.*

**Response:**

We thank the editor for this insight. We now emphasize the limited number of providers who routinely perform FGCS in the **Results**, **Discussion**, and **Conclusion** sections. Due to sample size constraints, subgroup analysis was limited, but we added a note suggesting future research explore this in greater detail.

**4. Clarify “Advertising”**

*What do you mean by "advertising"? Could you be more specific?*

**Response:**

We clarified this term in the **Methods** section. “Advertising” refers to public promotional activities such as online campaigns, clinic websites, and social media used by healthcare providers or clinics to promote FGCS services.

**5. Ethics Statement Location**

*Your ethics statement should only appear in the Methods section.*

**Response:**

Corrected. The ethics statement now appears only in the **Materials and Methods** section.

**6. Data Availability**

*All data must be made freely available.*

**Response:**

We have uploaded a de-identified version of the dataset as **Supplementary File S1**, in compliance with the journal’s data policy. We also updated the **Data Availability Statement** accordingly.

**Reviewer #1**

*Were all these physicians from the same geographic area? Socioeconomic status?*

**Response:**

We have clarified that all participants were based in **Iran**, and the majority practiced in **urban healthcare settings**. Unfortunately, detailed socioeconomic data were not collected, and this limitation is now acknowledged in the **limitation section**.

*The sociodemographic information of the physicians should be included...*

**Response:**

Thank you for your comment. Unfortunately, additional sociodemographic information for the physicians is not available in the dataset, as it was not collected during the study.

*Amount of training received and prior FGCS performed should be reported.*

**Response:**

These details were already partially reported. We now enhance this reporting in the **Discussion**, clearly stating the percentage with formal training and number of procedures performed

*Was this study only about cis-females or did it include trans-females?*

**Response:**

Thank you for this important point. We clarify in the **Methods** that the study focused solely on cisgender women. This clarification has been added explicitly.

*It is interesting to elaborate on the cultural aspects of a study made in a conservative religious environment and compare it to data in the literature.*

**Response:**

We appreciate this suggestion. We now expanded the **Discussion** to reflect the cultural and religious context of Iran and how this may affect healthcare providers’ attitudes. We also compared findings to those in more liberal or secular societies where FGCS is perceived differently.

*Conclusions and recommendations to the future need to be clearer.*

**Response:**

We revised the **Conclusion** and **Future Directions** sections to present clearer and more actionable recommendations, including calls for standardized training, ethical guidelines, and further subgroup analysis.

We hope these revisions meet the editorial and reviewer expectations and respectfully request reconsideration for publication. Thank you again for the opportunity to revise our work.

Sincerely,

Dr. Marjan Ghaemi, on behalf of all authors
