# Supplementary material for: Attitude and knowledge of healthcare providers towards female genital cosmetic surgery: A cross-sectional study
Source: PLoS One. 2026 Mar 20;21(3):e0343634. doi: 10.1371/journal.pone.0343634 (PMC13004357; doi:10.1371/journal.pone.0343634)
Supplement: Attachment — Submitted filename: response.docx [file pone.0343634.s003.docx]

Dear Dr. Ghaemi,

We've checked your submission and before we can proceed, we need you to address the following issues:

1. We note that there were multiple versions of Response to Reviewer in your submission's file inventory. We have removed the older file(s), retaining the most recent version(s) for editorial review. Please double check your submission file inventory and let us know if any files appear to be outdated or absent.

We've returned your manuscript to your account. Please resolve these issues and resubmit your manuscript within 21 days. If you need more time, please email the journal office at plosone@plos.org. We are happy to grant extensions of up to one month past this due date. If we do not hear from you within 21 days, we will withdraw your manuscript.

Response: Thank you for your message regarding my submission. I have reviewed the file inventory in my account and confirm that the most recent version of the Response to Reviewer document is correctly retained. No outdated or absent files remain in the submission record.I appreciate your assistance in ensuring the submission materials are in order. I will proceed with resubmitting the manuscript within the required timeframe.Please let me know if any further clarification or documentation is needed from my side.

Sincerely,
